# Risk Assessment of Microplastics in Humans: Distribution, Exposure, and Toxicological Effects

**DOI:** 10.3390/polym17121699

**Published:** 2025-06-18

**Authors:** Yifei Li, Wei Ling, Jian Yang, Yi Xing

**Affiliations:** 1 School of Energy and Environmental Engineering, University of Science and Technology Beijing, Beijing 100083, China; lyf_33053085@163.com (Y.L.); b20200081@xs.ustb.edu.cn (W.L.); 2 College of Chemistry and Environmental Engineering, Shenzhen University, Shenzhen 518060, China

**Keywords:** microplastics, humans, trend factor, meta-analysis, machine learning, cell viability

## Abstract

Microplastics are widely present in the environment, and their potential risks to human health have attracted increasing attention. Research on microplastics has exhibited exponential growth since 2014, with a fast-growing focus on human health risks. Keyword co-occurrence networks indicate a research shift from environmental pollution toward human exposure and health effects. Additionally, Trend Factor analysis reveals emerging research topics such as reproductive toxicity, neurotoxicity, and impacts on gut microbiota. This meta-analysis included 125 studies comprising 2977 data samples. The results demonstrated that cytotoxicity in experimental systems was primarily concentrated in Grade I (non-toxic, 62.8%) and Grade II (mildly toxic, 27.6%). Notably, inhibitory effects on cells were significantly enhanced when microplastic concentrations exceeded 40 μg/mL or particle sizes were smaller than 0.02 μm. The Gradient Boosting Decision Tree (GBDT) model was applied to predict cell viability, achieving an R^2^ value of 0.737 for the test set and a classification accuracy of 81.5%. Furthermore, reproductive- and circulatory-system cells exhibited the highest sensitivity to microplastics, whereas connective-tissue cells had the lowest survival rates. The study also identified an overuse of polystyrene (PS) polymers and spherical particles in experimental designs, deviating from realistic exposure scenarios.

## 1. Introduction

Concern regarding microplastics was first raised in 1972 [1], and the term “microplastics” was formally defined in 2004 [2]. In 2022, microplastics were detected in human blood for the first time, raising concerns about potential health risks [3]. Microplastics are generally defined as plastic particles smaller than 5 mm in diameter, encompassing both primary microplastics manufactured directly and secondary microplastics formed by the degradation of larger plastic debris [4]. Microplastic pollution has now become ubiquitous in the global environment, with traces identified even in the most remote regions, such as the polar ice caps of Antarctica and the Arctic [5,6], the Himalayas [7], the Mariana Trench [8], and the Amazon rainforest [9]. Microplastics can enter the human body via food chains, bioaccumulation, and airborne transmission pathways [10,11]. Indeed, microplastics have been detected within human biological media such as blood [12], the placenta [13], and lung tissue [14]. These findings indicate that humans are already subject to microplastic intake through food, drinking water, and respiration. However, definitive conclusions regarding the specific health effects of microplastics in humans remain lacking. To date, most research has focused on the detection and transport of microplastics in the environment, while studies on their potential harm to human health are relatively limited.

Although epidemiological evidence is lacking, the development of animal and in vitro cell culture models has provided valuable insights into microplastic toxicity [15]. Zebrafish (Danio rerio) and marine medaka (Oryzias melastigma) have been widely employed in microplastic toxicology studies due to their ease of observation, and have demonstrated biotoxic effects, including cytotoxicity and genotoxicity [16,17,18]. For investigations pertinent to human health, rats and mice are commonly used as mammalian exposure models in microplastic toxicity research [19,20]. These studies have confirmed that microplastic exposure induces biotoxic effects in mammals, as evidenced by histopathological analysis, assessment of inflammatory biomarkers, and evaluation of gene expression and functional pathways. Moreover, co-exposure to microplastics and other pollutants can produce synergistic toxicities [21,22]. In contrast to animal models, in vitro cell culture systems under microplastic exposure enable the quantitative evaluation of direct effects and elucidation of underlying mechanisms.

Various human- and animal-derived cell culture models—including the intestinal epithelial cell line Caco-2, the respiratory epithelial cell line BEAS-2B, and the renal cell line HEK293—have been employed to evaluate microplastic-induced cytotoxicity and underlying mechanisms [23,24]. At the cellular level, exposure to microplastics induces inflammatory responses and oxidative stress, significantly upregulating the expression of pro-inflammatory cytokine genes such as IL-6 and IL-8 [25]. The stress responses triggered by different microplastic types also vary among cell lines: polyethylene (PE) particles markedly increase reactive oxygen species (ROS) levels in the human glioma cell line T98G, but exert negligible effects in the cervical epithelial cell line HeLa, whereas polystyrene (PS) particles elevate ROS in both cell types [26]. Conversely, some studies report no acute cytotoxicity at low doses or within specific particle size ranges, suggesting that toxicity depends on the exposure concentration, particle size, and duration of exposure [27]. Given the multifactorial nature of these influences, researchers have introduced machine learning into microplastic toxicity research to handle complex datasets and predict biological hazards. For instance, Liu et al. and Ferreira et al. employed machine learning-driven QSAR models and meta-analysis approaches to investigate microplastic effects in BEAS-2B and Caco-2 cells, respectively, demonstrating that such methods can effectively predict microplastic impacts on cellular models [28,29]. However, comprehensive investigations into how different cell types respond to microplastic exposure remain lacking.

To address the current fragmentation and lack of systematic evaluation in studies of microplastic impacts on human health, this work employs both a bibliometric/meta-analysis approach and data-driven modeling. First, experimental data from human-relevant microplastic studies were collated via bibliometric analysis and meta-analysis to quantify the relationships between microplastic type, particle size, concentration, exposure duration, and cell viability across cell lines of diverse origins. Second, these data were used to train and evaluate multiple machine learning models for predicting cellular viability responses. The study particularly compares sensitivity differences among cells from different tissue sources and assesses how common polymer chemistries, particle morphologies, and viability assay methods influence measured cell viability. Finally, the paper discusses existing limitations in the field and outlines directions for future research.

## 2. Materials and Methods

**Figure 1:**
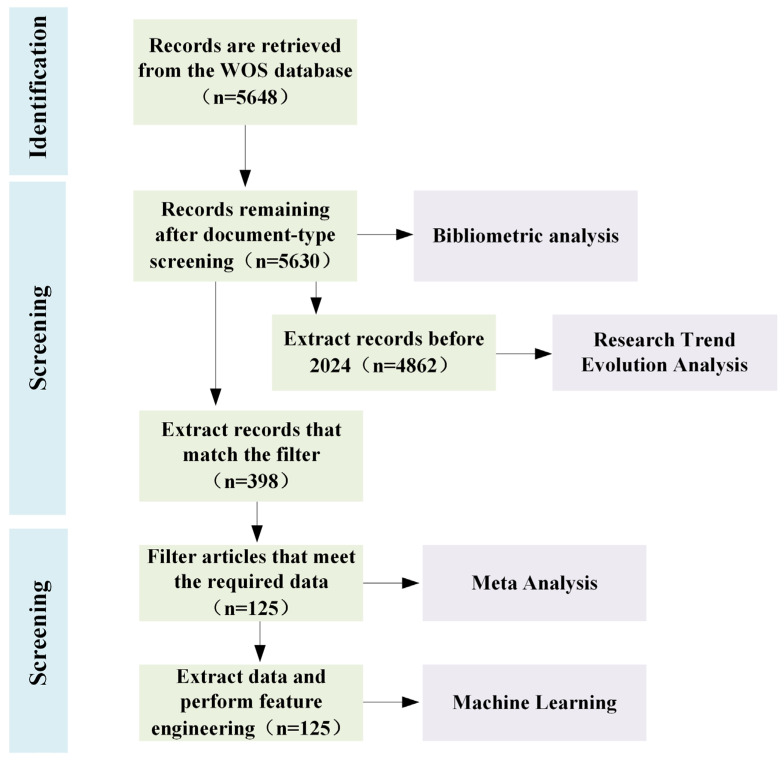
Flow diagram of data screening.

The workflow of the present study, grounded in existing research, is illustrated in Figure 1. The data screening process takes inspiration from the meta-analysis of microplastic cellular exposure conducted by O.G. et al. [29]. Detailed analytical procedures are provided in Section 2.1 and Section 2.2.

### 2.1. Bibliometric Analysis and Topic Evolution

In the bibliometric analysis of the literature on the impacts of microplastics on human health, the terms (“microplastic” OR “microplastics”) and “human” were used as search keywords in the Web of Science Core Collection database. All editions were included, and the search field was set to “Topic.” A co-occurrence network of author keywords appearing with a frequency greater than 23 was constructed and analyzed using VOSviewer^®^ (version 1.6.20) [30,31]. The annual number of publications, categorized by document type, was tabulated, and the overall publication trend from 2008 to 2024 was modeled via polynomial fitting in OriginPro 2025. Author keywords were standardized—merging synonyms and removing duplicates—using the Co-Occurrence 20.6 software (COOC 20.6) [32]. Records lacking author keywords but containing “Plus Keywords” were analyzed using those Plus Keywords. The Trend Factor (T) was employed to assess the temporal evolution of keyword usage, calculated as follows [33,34]:

(1) $$F_{R}=1000\times \frac{\sum_{2022}^{2024}f_{i}}{\sum_{2008}^{2021}N_{i}}$$

(2) $$F_{P}=1000\times \frac{\sum_{2008}^{2021}f_{i}}{\sum_{2008}^{2021}N_{i}}$$

(3) $$T=\log\left(\frac{F_{R}}{F_{P}}\right)$$

Here, *F*_R_ and *F*_P_ denote the frequencies of keyword occurrences in the recent three years (2022–2024) and the preceding period (2008–2021), respectively. In these expressions, *F*_R_ is the number of times the keyword appears in year *i*, and *F*_P_ is the total number of publications in year *i*. The resulting distribution of TTT values was visualized as a bubble chart using OriginPro 2025.

### 2.2. Meta-Analysis

To quantitatively assess the effects of microplastics on cells, the terms (“microplastic” OR “microplastics”) AND (“cell”) AND (“human”) were used to retrieve relevant articles from the Web of Science Core Collection, with the search cutoff date of 18 April 2025. Only studies employing human, rat, or mouse cell lines were included; data from other cell types (e.g., fish or avian cells) were excluded from this analysis. Exposure concentrations were standardized to μg/mL or equivalent units, whereas studies reporting exposure in particles/mL were not considered. Particle size was defined by the mean diameter of the microplastics, and polymer types were classified by single-polymer composition, encompassing both aged and modified polymers. In this paper, MPs and NPs represent the particle counts of microplastics and nanoplastics, respectively.

### 2.3. Machine Learning Analysis

For machine learning analysis, all experimental parameters—microplastic concentration, particle size, and exposure time—were standardized to units of μg/mL, µm, and h, respectively. Other categorical features were defined as follows: biological origin of cells (human, rodent); cell status (normal, tumor); organ-system classification (skeletal, respiratory, muscular, urinary, immune, endocrine, integumentary, nervous, reproductive, digestive, circulatory, other); cell morphology (stem/progenitor cells, muscle cells, connective-tissue cells, immune cells, epithelial cells, neural cells); microplastic polymer type (PS, modified PS, aged PS, non-PS, aged non-PS); microplastic shape (fragment, sphere, particle, other); and viability assay method (CCK-8, MTS, MTT, other methods). Prior to model development, all categorical variables were one-hot encoded. Cell viability values were categorized into toxicity grades according to established standards [35,36]: Grade I (non-cytotoxic, viability > 90%), Grade II (slight, 60% ≤ viability ≤ 90%), Grade III (moderate, 30% ≤ viability < 60%), and Grade IV (severe, viability < 30%). GBDT, CatBoost, and LightGBM models for regression and classification analyses were implemented using SPSSPRO 1.1.29 (www.spsspro.com). The data were split with a 0.9 train–test ratio, randomly shuffled, and evaluated via five-fold cross-validation.

## 3. Results and Discussion

### 3.1. Trends in Microplastic Research Related to Human Health

**Figure 2:**
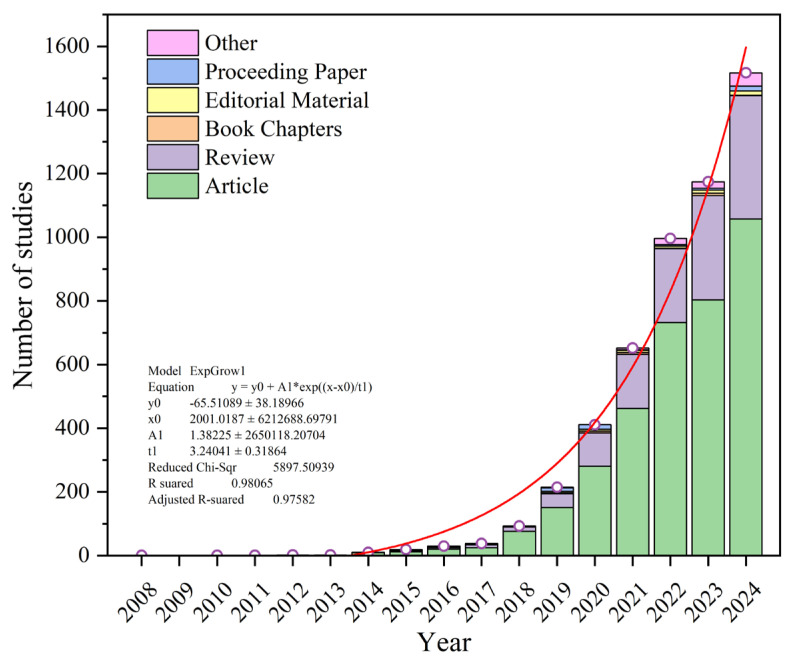
The temporal trend in the number of publications on microplastic impacts on human health.

A total of 5648 records were retrieved. After excluding items classified as “Publication With Expression Of Concern,” “Retracted Publication,” “Bibliography,” “News Item,” and “Expression Of Concern,” 5630 records remained. The time span covered 1 January 2000 to 18 April 2025. All records were categorized by primary document type (e.g., Article, Review) and by secondary classification (e.g., Meeting Abstract, Letter) and then arranged chronologically, as shown in Figure 2. The change in the total number of publications from 2008 to 2024 was fitted using a polynomial model. The results reveal that the overall trend exhibited exponential growth beginning in 2014, indicating a steadily increasing research interest. Articles and reviews constituted the two predominant publication formats, with reviews showing a pronounced rise between 2022 and 2024 in parallel with the overall publication increase. This pattern is consistent with previous trends in microfiber research [37]. Moreover, the rate of increase in human-related microplastic studies outpaced that of general microplastic research, suggesting that the human health–focused theme has received greater attention than studies of the pollutant itself.

**Figure 3:**
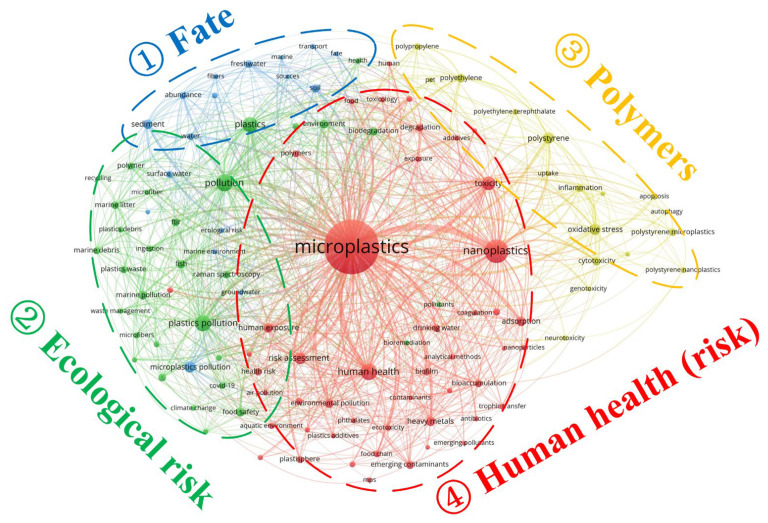
Co-occurrence network of author keywords for research on microplastic impacts on human health (keyword frequency > 23); node size is proportional to keyword frequency.

Furthermore, the interrelationships among author keywords were analyzed via a co-occurrence network (Figure 3). The analysis revealed that all topics cluster around “microplastics” in four primary modules. Module 1 (blue cluster) addresses the environmental “fate” of microplastics, illustrating their transport from “water” (freshwater/marine) into “sediment” or “soil.” In our previous study [38], we observed bidirectional transport of microplastics between aquatic and terrestrial compartments, with estuarine inputs delivering microplastics to the ocean. This module thus concerns the forms in which microplastics occur in the environment and their migration pathways driven by human activities. Module 2 (green cluster) captures the resulting “pollution”: in the “marine environment,” “fish” ingest microplastics via “ingestion,” leading to “ecological risk.” Our meta-analysis demonstrated that fish habitat influences microplastic ingestion levels, with benthopelagic species exhibiting abundances of 15.81 MPs per individual, compared to 10.95 and 8.12 MPs per individual in demersal and pelagic species, respectively [39]. Furthermore, our earlier review confirmed that environmental microplastic pollution predominantly takes the form of microfibers [37]. Module 3 (orange cluster) further delineates the polymer types responsible for pollution, including polyethylene (PE), polypropylene (PP), polyethylene terephthalate (PET), and polystyrene (PS). Module 4 (red cluster)—the group most closely connected to “microplastics/nanoplastics”—indicates that these “emerging pollutants/contaminants” threaten “human health (risk)” through “human exposure.” This exposure pathway involves “drinking water” and the “food chain,” resulting in “trophic transfer” and “bioaccumulation.” Overall, although human-related microplastic research spans topics such as sources, distribution, and morphology, its principal focus remains the assessment and elucidation of risks to human health and safety.

**Figure 4:**
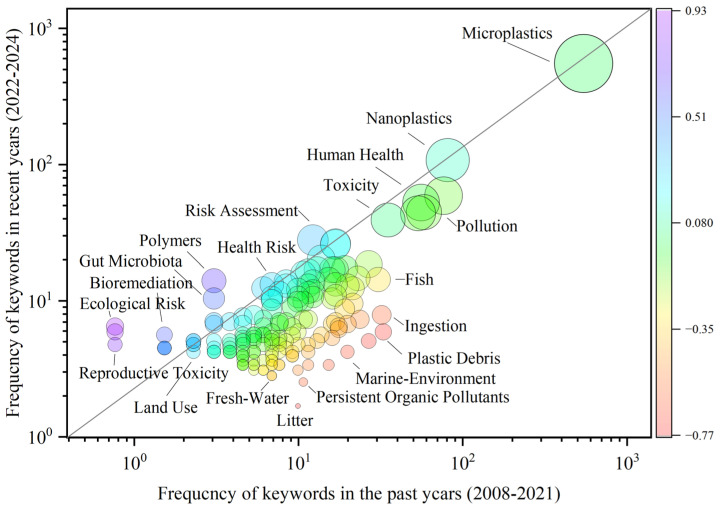
Evolutionary analysis of trends in research on microplastic impacts on human health (T > 0 indicates that the keyword has exhibited an upward trend over the past three years; T < 0 indicates a downward trend).

Trend Factor analysis was employed to assess the shifts in research hotspot trends over the recent three-year period (2022–2024) relative to the preceding fourteen years (2008–2021), as shown in Figure 4.

Analysis indicates that early research concentrated on microplastic ingestion by fish in aquatic environments (marine and freshwater), leading to environmental pollution, as well as the synergistic toxicity between microplastics and persistent organic pollutants. These themes correspond to Modules 1 and 2 identified in the clustering analysis. In contrast, current research hotspots on micro- and nanoplastics have shifted toward human health and environmental risk assessment. As shown in Figure 4, there is a rising focus on the effects of polymers on gut microbiota during bioaccumulation, as well as ecological risk and genotoxicity. These topics align with Modules 3 and 4 of the clustering analysis. Overall, these findings demonstrate that, with the deepening of research, studies on the impacts of microplastics have moved beyond source tracing and ingestion by environmental biota. Given that no standardized methods for large-scale removal of environmental microplastics are yet available [40], the threat to human health posed by microplastics is likely to persist in the short term. Therefore, based on existing insights, there is an urgent need for more in-depth investigation into the effects of microplastics on human health to accurately assess risks and develop strategies for their mitigation.

### 3.2. Analysis of Research on Microplastic Impacts on Human Health

#### 3.2.1. Pathways of Human Exposure to Microplastics

Human exposure pathways for microplastics include ingestion, inhalation, dermal contact (e.g., via cosmetics and personal care products), intravenous introduction during medical procedures, and maternal–infant transfer [41,42,43,44]. Among these, inhalation and dietary intake are the two primary routes of microplastic entry into the human body [45]. Microplastics have been detected in commercially available soft drinks packaged in plastic bottles, aluminum cans, and glass bottles; based on an annual per capita consumption of 41.13 L, this route may result in an intake of 81–1609 particles per year [46]. The aging of packaging materials and high-temperature use exacerbate the release of micro- and nanoplastics into food, where they can reach levels of 10^4^ MPs and 10^7^ NPs [47]. Once ingested, microplastics transit through the gastrointestinal tract and are subsequently excreted in feces [48]. In contrast, respiratory exposure to airborne microplastics involves more complex sources and distributions. Simulations indicate that, compared to homes, subways, and workplaces, exposure on buses (17.3 ± 2.4 MPs/m^3^) leads to the highest deposition of microplastics in the body (approximately 220 MPs) [49]. The pulmonary distribution of inhaled microplastics depends on particle shape and size. In female rats, inhalation of 5 µm spherical polyamide micro- and nanoplastic particles resulted in deposition throughout all lung regions; although no pulmonary inflammation was observed, vascular dysfunction and elevated blood pressure were noted in the uterine vasculature [50]. ExDoM2 dosimetry modeling further revealed that larger fiber-shaped microplastics (diameter > 0.1 µm) preferentially deposit in the extrathoracic region, whereas smaller particles (diameter < 0.1 µm) predominantly lodge in the alveolar region [49]. Subsequently, most of these deposited microfibers (66.4%) are cleared by mucociliary action and transported to the esophagus. Additionally, microplastics entering the body may be eliminated via secretions such as tears and meibum [51].

Although the overall contribution of other exposure routes to systemic microplastic intake is relatively limited, these pathways introduce particles directly into the circulatory system, and therefore warrant increased scrutiny. Research indicates that the size threshold for direct penetration of the dermal barrier by microplastics is <100 nm [52]. Microplastics measuring 100–400 μm can traverse the placental barrier via the placenta–umbilical cord blood–meconium route, enabling vertical transmission from mother to fetus and posing intergenerational health threats [53]. During gestation, maternally ingested polystyrene nanoplastics (PS nanoplastics) can be transferred to fetal brain tissue through breast milk, leading to neurofunctional disruption and cognitive deficits in offspring [54]. Of particular importance, invasive medical procedures employing plastic-based devices (e.g., percutaneous coronary intervention) have been shown to introduce microplastics into patient blood, with postoperative microplastic concentrations reaching up to 18.8 times the levels at baseline [55]. The incorporation of filtration systems may be an effective strategy to reduce microplastic entry into patients. Precision infusion systems (PISs) equipped with built-in filters can reduce microplastic concentrations in infusates (2.91 ± 3.91 MPs/L) by over 50% and substantially diminish microfiber abundance [56]. However, it should be noted that even filtered fluids still contain small microplastics (≤110 μm) capable of entering the bloodstream. Consequently, intravenous infusion represents an active iatrogenic exposure route whose risk may be underestimated, particularly among chronic patients receiving long-term infusion therapy. The size distribution of microplastics is directly related to filter efficacy; thus, improvements in medical device design—such as the implementation of nanometer-scale filtration membranes—could further reduce the risk of iatrogenic microplastic exposure.

#### 3.2.2. Distribution and Migration of Microplastics in the Human Body

Several studies have confirmed that the presence of microplastics in human blood is a globally pervasive issue [57]. In a cross-sectional study of healthy Korean adults, microplastics were detected in 88.9% of participants’ blood (mean concentration 4.2 MPs/mL), predominantly polystyrene (PS, 1.7 MPs/mL) and polypropylene (PP, 1.4 MPs/mL), with concentrations significantly associated with frequent use of plastic food containers [58]. Similarly, a study in the Netherlands employed pyrolysis–gas chromatography/mass spectrometry (Py-GC/MS) to quantify polyethylene (PE), polyethylene terephthalate (PET), and polystyrene (PS) in the blood of 77% of adult subjects, yielding an average total concentration of 1.6 µg/mL [3]. The widespread detection of microplastics in blood suggests they may disseminate via the circulatory system to various organs, underscoring the need to assess long-term bioaccumulation. Indeed, investigations have demonstrated that ingested micro- and nanoplastics accumulate in the kidneys and liver, translocate to the brain through both hematogenous and neural pathways, and reach peak abundance in cerebral tissue [59,60]. In animal models, nanoplastics entering via the gastrointestinal route are distributed to the heart through the bloodstream, activate the TNF-α/NF-κB and p38/MAPK signaling pathways, and elicit inflammatory responses, oxidative stress, myocardial fibrosis, and apoptosis [61].

The ability of microplastics to penetrate physiological barriers is closely related to their particle size and surface functional-group characteristics [62]. Even for a single polymer type, environmental aging—such as ultraviolet irradiation and air exposure—can alter surface chemistry, molecular weight, and morphology [63]. Translocation of microplastics across biological barriers may give rise to more complex systemic toxicity. In particular, microplastics can cross the blood–brain barrier (BBB) and affect the central nervous system; with decreasing particle size, polystyrene nanoplastics (~200 nm) exhibit higher transendothelial transport and permeability than larger microplastics (1.0 µm) [64]. Inflammatory processes further exacerbate this penetration. Microplastics ranging from 5.5 to 26.4 µm have been detected in the human olfactory bulb, suggesting their potential to induce neurotoxicity upon reaching brain tissue [65]. Similarly, microplastics traverse the blood–testis barrier (BTB) via endocytic and exocytic pathways in Sertoli cells, and inflammation-induced increases in endothelial permeability further enhance this process [66]. Even biodegradable plastics can pose physiological risks: in male mice, polylactic acid (PLA) micro-/nanoplastics compromise BTB integrity and trigger oxidative stress through mitochondrial dysfunction, leading to reproductive toxicity [67].

#### 3.2.3. Effects of Microplastic Exposure on Human Health

Although no definitive causal link between microplastics and specific human diseases has been established, observational studies have identified strong associations between the presence of microplastics in various tissues and certain health conditions: polyethylene microplastics in the brain and neurodegenerative disorders; PVC and PA66 microplastics in blood and extracranial artery stenosis (ECAS); PS, PVC, PET, PMMA, POM, and PP microplastics in cirrhotic liver tissue; PE microplastics in tear fluid and meibum with dry eye disease (DED); and inhaled microplastic particulates in the lung with pneumoconiosis [51,60,68,69,70]. Microplastic clinical effects are both dose- and time-dependent: chronic low-dose exposure may lead to persistent inflammation and metabolic dysregulation, whereas acute high-dose exposure (e.g., during medical procedures) can precipitate thrombosis and other acute events [71,72]. Proposed mechanisms of microplastic toxicity include physical damage (rough particle surfaces can disrupt cellular membranes) [73], chemical additive release (leaching of plasticizers and flame retardants from the particle matrix) [74], and synergistic effects (enhanced toxicity due to co-adsorbed environmental contaminants, such as PAHs, desorbing under gastrointestinal conditions) [75]. Environmental aging processes—such as UV weathering and interaction with airborne contaminants—alter microplastic surface chemistry, molecular weight, and morphology, thereby amplifying neuroinflammatory responses in brain microglia [63]. Moreover, combined exposure to microplastics and other pollutants (e.g., methylmercury, PCBs) may produce additive or synergistic toxic effects, underscoring the need for bioaccumulation-based risk assessment frameworks [76,77]. As vectors for heavy metals and organic toxins, microplastics can act via a “Trojan horse” mechanism, enhancing contaminant bioavailability—particularly under the low-pH conditions of the stomach—and thus exacerbating toxicity [78].

Microplastics and nanoplastics have drawn considerable attention due to their potential neurotoxic effects on the central nervous system. Studies indicate that the infiltration of microplastics/nanoplastics into brain tissue can elicit inflammation, oxidative stress, and mitochondrial dysfunction, subsequently causing neurobehavioral abnormalities and cognitive deficits [79,80,81]. Acute and subchronic exposure to polystyrene microplastics (PS microplastics) has also been shown to induce anxiety-like behavior in mice and to reduce neuronal populations in the dentate gyrus (DG) and Cornu Ammonis (CA) regions of the hippocampus, accompanied by histopathological evidence of neuronal damage [82]. Beyond direct cell–particle interactions, microplastics can inflict indirect damage via vascular mechanisms. PS particles (0.08–5 µm) entering the bloodstream are phagocytosed by neutrophils and macrophages, which may then form microthrombi that occlude cortical capillaries and precipitate neurological dysfunction [83]. Intriguingly, inhaled PS microplastics—although not directly accumulating in cerebral tissue—can provoke neurotoxicity through the lung–brain axis: pulmonary microbiota dysbiosis generates lipopolysaccharide (LPS) that activates microglial M1 polarization, ultimately leading to cognitive impairment [84]. In models of Alzheimer’s and Parkinson’s disease, MP exposure promotes neurodegeneration via gut microbiota dysbiosis and immune activation, underscoring the pivotal role of the gut–brain axis in MP-induced neurotoxicity [85]. Animal experiments have further demonstrated that MP-induced neural injury can alter social behavior: chronic PS-MP exposure in dams leads to social novelty preference deficits in offspring, despite no significant changes in affective or general cognitive function [86]. Additionally, exposure to polyethylene (PE) microplastics has been shown to perturb the murine gut microbiome and induce autism spectrum disorder-like behaviors, highlighting potential developmental neurotoxicity [87].

Beyond neurotoxicity, microplastic exposure can induce a spectrum of metabolic disturbances ranging from subcellular to systemic scales. Mitochondria—the cellular organelles responsible for energy production—also serve as targets for microplastics; microplastic-induced oxidative stress inhibits ATP synthesis and triggers cell death [88]. While oxidative stress is commonly observed in piscine microplastic exposure experiments, it does not always manifest under human cell exposure, suggesting the existence of more sophisticated defense and repair mechanisms in human tissues. In human brain vascular pericytes, exposure to polyethylene terephthalate (PET) particles did not elicit oxidative stress, but significantly impaired mitochondrial respiratory function after 3 days; mitochondrial efficiency subsequently recovered via compensatory responses [89]. The reversible nature of PET-induced mitochondrial dysfunction implies adaptive repair capacity, though whether repeated exposures produce cumulative damage remains to be determined. Similarly, in the reproductive system, oral administration of PET microplastics (1 µm, 0.01–1 mg/d) in male mice induced oxidative stress and testicular histopathology; these effects were reversed by N-acetylcysteine (NAC) through inhibition of the p38 signaling pathway [90]. Giant unilamellar vesicle (GUV) models further demonstrate that increased hydrogen-bond interactions between polystyrene microplastics and lipid bilayers disrupt phospholipid architecture and reduce membrane fluidity [62]. Animal studies also reveal that PS exposure perturbs the gut–liver axis and promotes insulin resistance, indicating broader metabolic derangements beyond isolated organ systems [91].

### 3.3. Meta-Analysis of the Effects of Microplastic Exposure on Cell Viability

#### 3.3.1. Effects of Microplastic Exposure Conditions on Cell Viability

Studies have demonstrated that, in cellular exposure assays with microplastics, cell viability is influenced by multiple factors, including the quantitative exposure parameters of the microplastics [92], the biological characteristics of the test cells [93], and the assay methodologies employed [94]. Microplastics can impact cells by activating apoptotic pathways, and their toxicity increases with longer exposure durations, higher concentrations, and smaller particle sizes [95]. Based on the above criteria, we performed a study-by-study screening of the literature on microplastic exposure in human cell models, ultimately selecting 125 publications comprising 2978 data points for statistical analysis.

**Figure 5:**
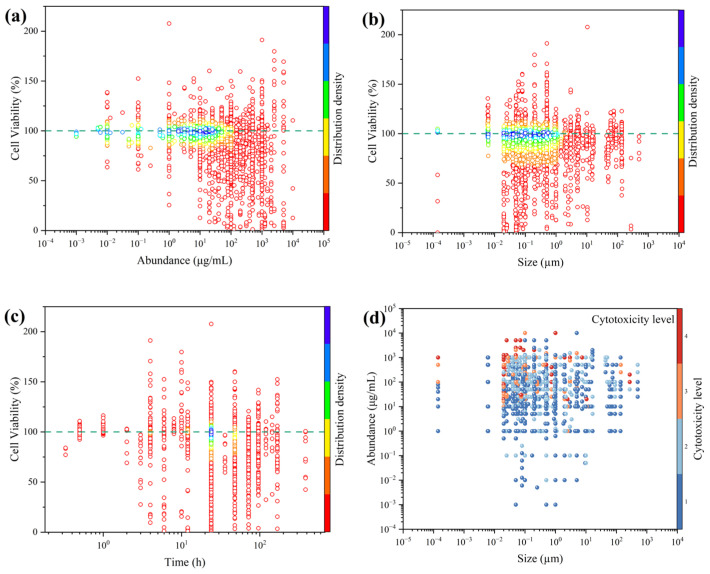
Distribution of the effects of key exposure parameters on cell viability: (**a**) concentration, (**b**) particle size, (**c**) exposure duration, and (**d**) combined concentration–size synergistic impact on cytotoxicity. (Each data point represents a data point in a study, where the colors in (**a**–**c**) represent the density distribution of the data points, with red to blue representing an increasing trend in the distribution concentration of the data points. The color in (**d**) represents the cytotoxicity level of the data points, with red to blue representing an increasing trend in the cytotoxicity level).

The effects of exposure concentration on cell viability were first evaluated (Figure 5a). The experimental concentrations spanned 0.001–10,000 μg/mL. Within the 1–40 μg/mL range, cell viability (CV) values clustered around 100%, indicating that microplastic exposure at these concentrations had minimal impact on cell viability. However, at concentrations above 40 μg/mL, CV exhibited a pronounced decline, suggesting that higher exposure levels elicit increased cytotoxicity. Because microplastics are insoluble in aqueous media and cellular internalization is a key step in their bioactivity, elevated concentrations likely promote greater particle uptake by cells [96]. Moreover, the higher probability of particle–cell contact at these concentrations may provoke stronger inflammatory responses, further reducing CV.

Under varying microplastic particle sizes (0.00014–500 μm), the distribution of cell viability (CV) is shown in Figure 5b. Within the 0.02–200 μm range, CV values exhibit a downward trend as particle size decreases. Relevant studies have found that, at the same exposure concentration, the cellular internalization of 50 nm and 500 nm microplastics is enhanced compared to that of 5000 nm microplastics [97]. However, CV also clusters near 100% under exposure conditions of 0.02–1 μm, indicating that cytotoxicity is not determined solely by particle size. Furthermore, by mapping cell viability values to toxicity grades, the combined effects of microplastic concentration and particle size on cytotoxicity were analyzed using a four-level classification scheme (Figure 5d). Within the current dataset, smaller particle size and higher concentration correlate with greater cytotoxicity, with the region around 0.02 μm and 1 250 μg/mL showing the highest risk. Additionally, exposure durations in the literature span 0.017–384 h, with 24 h being the most frequently used duration (49.6% of all data points). As illustrated in Figure 5c, CV values at 24 h likewise concentrate near 100%. Importantly, CV decreases with increasing exposure time. These trends in cell viability relative to exposure parameters are consistent with prior studies, yet our analysis uncovers a clear threshold effect: even as decreasing particle size generally reduces CV, values continue to accumulate around 100%. Therefore, it is essential to explore the synergistic mechanisms among these factors in order to accurately assess the impact of microplastics on cell viability.

#### 3.3.2. Effects of Cell Status on Viability Under Microplastic Exposure Conditions

**Figure 6:**
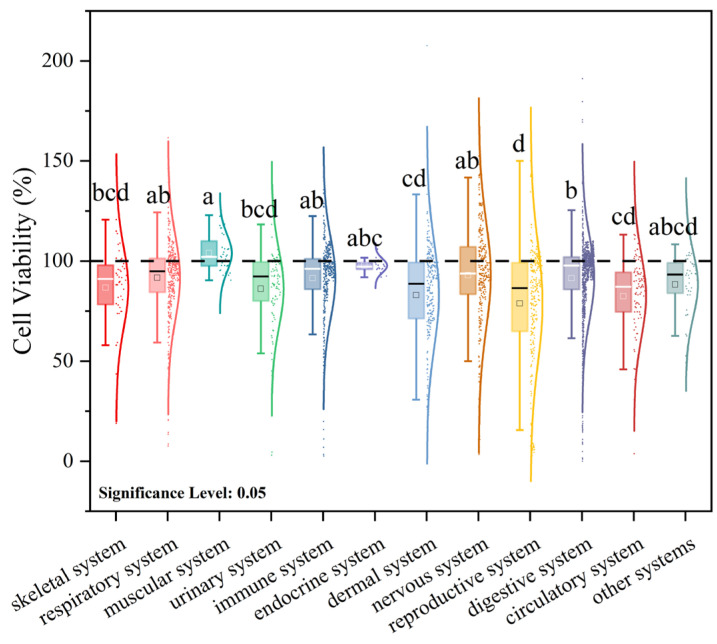
Effects of microplastic exposure on cell viability in cell lines derived from different organ systems.

In fact, beyond the influence of microplastic exposure parameters on cell viability (CV), the intrinsic characteristics of different cell lines—originating from distinct organisms and tissues—also modulate CV under identical exposure conditions. This likely reflects differential sensitivity to microplastic toxicity among cell types under the same exposure regimen [98,99,100]. The mean CV values by organ system are ranked as follows: muscular system (103.9 ± 9.1%) > endocrine system (97.7 ± 3.4%) > nervous system (92.9 ± 27.2%) > respiratory system (91.7 ± 20.9%) > digestive system (91.5 ± 20.6%) > immune system (91.4 ± 20.1%) > other systems (88.3 ± 16.1%) > skeletal system (86.7 ± 20.3%) > urinary system (86.1 ± 19.4%) > integumentary system (83.0 ± 25.8%) > circulatory system (82.5 ± 20.6%) > reproductive system (78.8 ± 30.1%) (Figure 6). Notably, although microplastics are known to induce neurotoxicity, neuronal cell lines exposed directly in vitro remain classified as Grade I (non-cytotoxic). This suggests that observed neurotoxic effects in vivo may be mediated predominantly via the lung–brain and gut–brain axes, rather than being the result of direct cytotoxicity. Conversely, cells of the skeletal, urinary, integumentary, circulatory, and reproductive systems exhibit greater suppression of viability under microplastic exposure. Of particular concern are cells of the circulatory and reproductive systems, which appear most susceptible to microplastic-induced adverse effects—potentially impairing systemic transport functions and contributing to reproductive toxicity.

**Figure 7:**
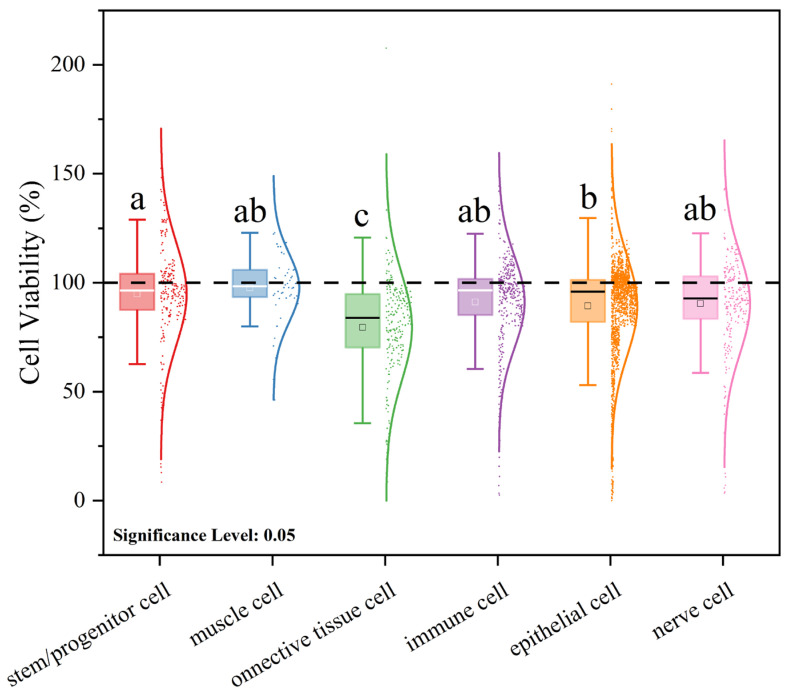
Effects of microplastic exposure on cell viability across different cell types.

Aside from system origin, the cell lines used in these experiments are typically classified into four principal tissue types based on morphology and function: epithelial cells, connective-tissue cells, muscle cells, and neural cells. In the present dataset, stem/progenitor cells and immune cells are treated as separate categories. The distribution of cell viability by cell type is shown in Figure 7.

The mean cell viability values are ranked as follows: muscle cells (97.6 ± 15.6%), stem/progenitor cells (94.9 ± 23.3%), immune cells (91.1 ± 21.0%), neural cells (90.4 ± 23.0 %), epithelial cells (89.3 ± 22.8%), and connective-tissue cells (79.5 ± 24.4%). These findings indicate that muscle cells, stem/progenitor cells, and immune cells maintain relatively high viability under microplastic exposure, whereas connective-tissue cells are most susceptible to cytotoxic effects. Bianchi et al. report that micro-/nanoplastic cytotoxicity is generally low, and varies according to cell type and particle composition [101]. Consistently with this, our results demonstrate that the average microplastic toxicity across different cell types remains within the slight-to-non-cytotoxicity range. It is important to note that cell viability, while a key indicator of microplastic toxicity, is not the sole determinant. Wang et al. observed that although polystyrene microplastics exert minimal effects on myoblast viability, they nonetheless induce reactive oxygen species (ROS) production and oxidative stress, thereby impairing muscle fiber regeneration [102]. Thus, comprehensive toxicity assessments should incorporate multiple endpoints. Similarly, stem/progenitor cells, despite experiencing ROS-mediated stress upon microplastic exposure, may leverage their high self-renewal capacity and multipotent differentiation potential to sustain viability relative to other cell types [103]. Furthermore, downregulation of inflammatory phenotypes in immune cells under microplastic challenge suggests activation of intrinsic repair mechanisms [104]. Overall, variability in cell-type sensitivity to microplastic-induced toxicity contributes to the observed differences in cell viability outcomes.

#### 3.3.3. Effects of Polymer State on Cell Viability Under Microplastic Exposure Conditions

**Figure 8:**
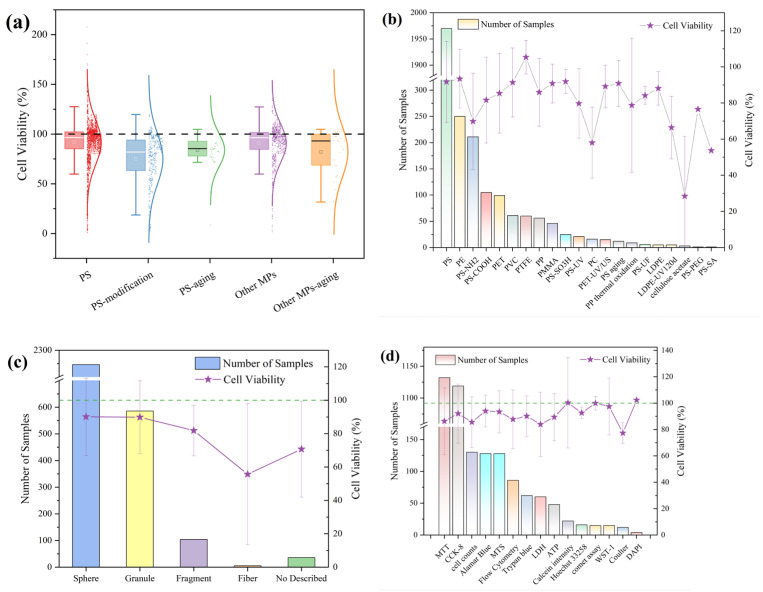
Effects of microplastic characteristics on cell viability (CV) distributions: (**a**) by surface properties, (**b**) by polymer type, (**c**) by particle morphology, and (**d**) by viability assay method.

The polymer composition and morphology of microplastics also exert differential effects on cells via variations in surface topography and functional groups [92,105,106]. Most current studies employ polystyrene (PS) as the model microplastic compound; some investigations have reported that PS exhibits comparatively high toxicity [99]. However, Lithner et al. ranked polyurethane (PUR), polyacrylonitrile (PAN), and polyvinyl chloride (PVC) as having hazard scores of 13,844, 12,379, and 10,001, respectively, whereas PS scored only 30 [107]. Similarly, Yuan et al. found that PS’s hazard score was comparable to that of polyethylene (PE) and polypropylene (PP), but lower than those of PUR, PAN, and PVC [108]. Consequently, PS may not adequately model the cellular impacts of more hazardous microplastic polymers. Moreover, environmental surveys indicate that the abundance of PS is generally lower than that of PE and PP, a pattern mirrored in human tissue samples, where PS is less prevalent among detected microplastics [60]. This discrepancy likely reflects the dominant use of PE- and PP-based plastics in food packaging, which generates corresponding micro-/nanoplastic particles upon wear and weathering [109]. Therefore, selecting PS as the sole exposure model may fail to represent the polymer types that are most relevant to environmental and human exposures. In our dataset, the means and distributions of cell viability under PS and non-PS microplastic exposures are similar, as shown in Figure 8a. This indicates that PS does not produce markedly greater cytotoxicity than other polymers; however, surface modification and weathering of microplastics significantly reduces cell viability, a trend also evident in Figure 8b. It is noteworthy that PS and PE are the most commonly studied polymers; despite differences in sample size, their impacts on cell viability are comparable, suggesting relatively small differences in cytotoxic potential between these two materials.

Similarly, current in vitro studies demonstrate a pronounced preference for spherical and granular microplastics, with spherical particles being the most frequently employed. As shown in Figure 8c, the effects of spherical and irregular microplastics on cell viability are nearly identical, indicating minimal shape-dependent differences in cytotoxic potential. In response to concerns about primary microplastics in the form of microbeads, numerous countries have enacted legislation to limit their environmental release [110]. In contrast, secondary microplastics generated from food packaging and ambient air largely exist as fragments and fibers [111,112]. Whether these experimental models accurately reflect real-world exposure scenarios therefore requires further scrutiny. Moreover, as depicted in Figure 8d, cell viability assays vary across studies. Even within a single investigation, different viability assays can produce divergent results under identical exposure conditions [94,113]. Although such assays are widely used to quantify post-exposure cytotoxicity, the impact of methodological choice remains poorly characterized. Additionally, heterogeneity in methodological parameters—such as reporting units (particles/mL versus μg/mL) and selected effect endpoints—increases the difficulty of cross-study comparisons. Establishing unified, standardized protocols is therefore essential to correct for inter-study variability and ensure the comparability of results. In addition to the cell viability assays mentioned above, colony-forming assays, though time consuming, may give a more complete picture of cytotoxicity, as they require cell division in addition to metabolic activity.

#### 3.3.4. Comprehensive Impact Analysis

**Figure 9:**
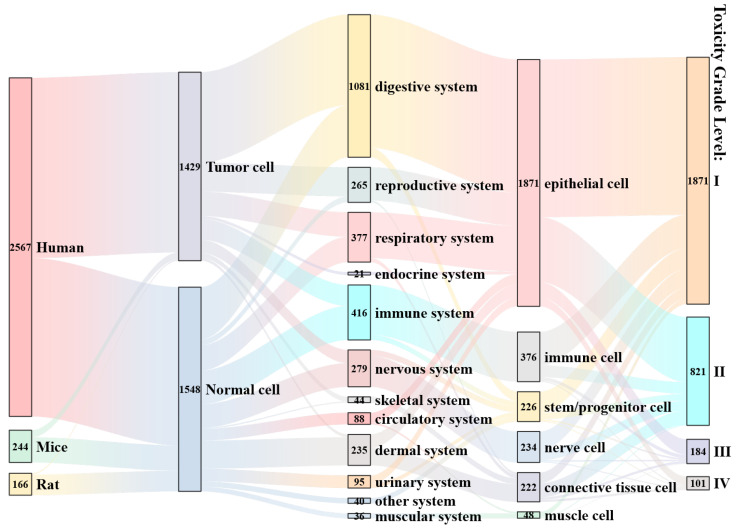
A Sankey diagram illustrating the impact of various classification parameters on cellular toxicity.

Current research has primarily concentrated on specific materials and model systems, introducing certain biases, as depicted in Figure 9. In terms of model selection, the majority of studies utilize human-derived cell lines, with a pronounced emphasis on digestive-system epithelial cells, such as Caco-2 [92] and HepG2 [94], including both tumor-derived and non-tumor phenotypes. Data coverage across organ systems and cell types is markedly uneven; for example, only 21 data points originate from endocrine-system cells. When classified by cytotoxicity grade, the bulk of findings fall into Grade I (62.8%) and Grade II (27.6%), indicating relatively low effects of microplastic-induced toxicity on cell viability under current experimental conditions. It is noteworthy, however, that although epithelial cells predominantly exhibit Grade I and II responses, they also account for the largest share of Grade III and IV toxicity observations.

**Figure 10:**
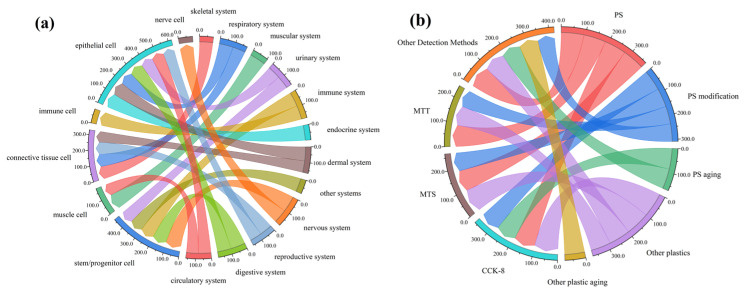
Chord diagrams illustrating interrelationships among classification parameters: (**a**) interaction between organ systems and cell classifications, and (**b**) interaction between assay methods and microplastic classifications.

Based on the foregoing analysis, the combined effects of different factors on cell viability were assessed using chord diagrams (Figure 10). Two factor pairs were examined: (1) organ-system versus cell classification, and (2) assay method versus polymer type. The results indicate that, for a given cell type, the average viability varies only marginally across different organ systems. Similarly, under different assay methods, viability differences across various polymer exposures are also minimal. These findings suggest that the interactions between these factor pairs do not substantially influence the overall mean cell viability.

In summary, current evidence indicates that microplastic-induced effects on cell viability do not follow a simple linear relationship. Some studies report an increase in cell viability, while others observe a decrease. Future research should more comprehensively incorporate factors such as polymer aging state, synergistic interactions with co-pollutants, and chronic low-dose exposures in order to elucidate the mechanistic basis of microplastic toxicity and inform environmental risk assessments. Apart from acute toxicity, future studies should also include endpoints such as subchronic and chronic toxicity indicators, cross-organ toxicity assessment, and in-depth analysis of molecular mechanisms. In addition to broadening the diversity of polymer types and cellular models, forthcoming studies must emphasize methodological harmonization—such as standardizing exposure-dose units and unifying biomarker selection—and adopt multi-endpoint assessment frameworks to accurately reflect the toxicological profiles arising under realistic, heterogeneous environmental exposure scenarios.

### 3.4. Machine Learning Analysis of the Effects of Microplastic Exposure on Cell Viability

Based on the parameter selections described above, machine learning approaches were employed to evaluate cell viability (CV) by constructing predictive models. Three gradient-boosting tree algorithms—GBDT, CatBoost, and LightGBM—were selected for both regression and classification tasks. In the regression of cell viability, all three models achieved a good fit, with subtle performance differences. The GBDT model attained the highest coefficient of determination on the test set (R^2^ = 0.737), compared to 0.723 for CatBoost and 0.702 for LightGBM (Table 1). Likewise, in the classification of cell viability grades, GBDT delivered the highest test-set accuracy (81.5%), followed by CatBoost (79.5%) and LightGBM (75.8%) (Table 2). Overall, the GBDT model slightly outperformed the others in predicting cell viability under microplastic exposure, indicating its stronger ability to capture complex non-linear relationships. Nonetheless, all three models demonstrated effective identification of the key factors influencing cell viability.

#### 3.4.1. Regression Analysis of Cell Viability Under Microplastic Exposure

**Figure 11:**
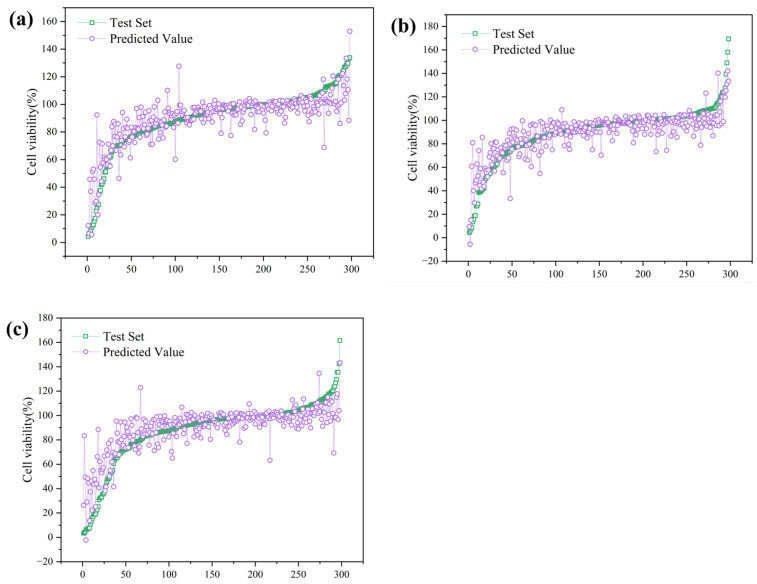
The test-set regression prediction results for the three machine learning models (**a**) GBDT, (**b**) CatBoost, and (**c**) LightGBM.

On the training set, the GBDT model attained an R^2^ of 0.87—substantially higher than its R^2^ of 0.737 on the test set—indicating a degree of overfitting. In contrast, CatBoost achieved an R^2^ of 0.806 on the training set and 0.723 on the test set, demonstrating superior generalization. LightGBM, meanwhile, exhibited a training R^2^ of 0.759, which declined to 0.702 on the test set. The test-set predictions are shown in Figure 11. These discrepancies likely stem from intrinsic algorithmic characteristics. CatBoost’s specialized gradient-boosting strategy reduces information leakage and overfitting when handling categorical variables, resulting in balanced training performance and robust test generalization [114]. Conversely, LightGBM’s leaf-wise tree growth can produce overly deep structures when data are limited, making it sensitive to noise and outliers, which aligns with its larger train–test performance gap. GBDT strikes a balance between efficiency and regularization, employing cross-validation to tune hyperparameters and mitigate overfitting; this enabled it to achieve the highest test R^2^ and overall accuracy in our study. Finally, the extensive one-hot encoding of experimental variables may have affected model behaviors: while the resulting high-dimensional feature space increased learning complexity, CatBoost did not fully leverage its native handling of categorical features, possibly constraining its relative performance.

When compared with the existing literature, our model performance is broadly consistent, yet exhibits minor differences. For example, Liu et al. developed QSAR models to predict microplastic cytotoxicity in human bronchial epithelial BEAS-2B cells, evaluating six machine learning algorithms; their XGBoost model achieved a test-set R^2^ of 0.93, markedly higher than our GBDT R^2^ of 0.74 [28]. This discrepancy likely reflects differences in dataset scope and feature selection: Liu et al. focused on a single cell line and five common microplastics, which simplified model learning and yielded a higher fit, whereas our dataset spans multiple cell types, polymer chemistries, morphologies, concentrations, and exposure durations, introducing greater heterogeneity and resulting in a somewhat lower maximal R^2^. Similarly, Ferreira et al. used a random forest model to predict micro- and nanoplastic cytotoxicity in Caco-2 cells, reporting a test-set R^2^ of 0.81—slightly above our results—but their analysis was based on 320 literature-derived data points from a single, rigorously defined cell model [29]. Under such controlled conditions, models more readily attain uniformly high performance. The fact that we achieved an R^2^ near 0.75 across a broader range of in vitro models and microplastic characteristics underscores the competitiveness of gradient-boosting approaches like GBDT and CatBoost for integrating and predicting complex toxicological outcomes.

Moreover, future investigations could benefit from insights gained in machine learning studies of other pollutant cytotoxicity. For instance, one meta-analysis applied decision tree and random forest algorithms to synthesize cytotoxicity data for plant extract-derived silver nanoparticles (Ag-nanoplastics) [115]. In that study, a single decision tree model achieved a regression R^2^ of approximately 0.97, outperforming the random forest model’s R^2^ of approximately 0.87. The authors attributed this to the decision tree’s use of threshold-based splits to isolate key predictive factors, thereby mitigating overfitting, whereas the random forest’s aggregation of more complex relationships reduced its generalization capacity. This finding highlights the necessity of aligning model complexity with data complexity: when sample sizes are limited or signal-to-noise ratios are low, overly sophisticated ensemble models may capture noise rather than true signals. In our work, we similarly observed that, after comprehensive hyperparameter optimization, ensemble methods such as GBDT and CatBoost delivered the best overall performance, while simpler models sometimes demonstrated greater robustness. Although specialized models tailored to a single material or cell line can yield superior results, our analysis—encompassing diverse microplastic types and cellular contexts—demonstrates the practical utility of gradient-boosting approaches for predicting complex toxicity outcomes.

**Figure 12:**
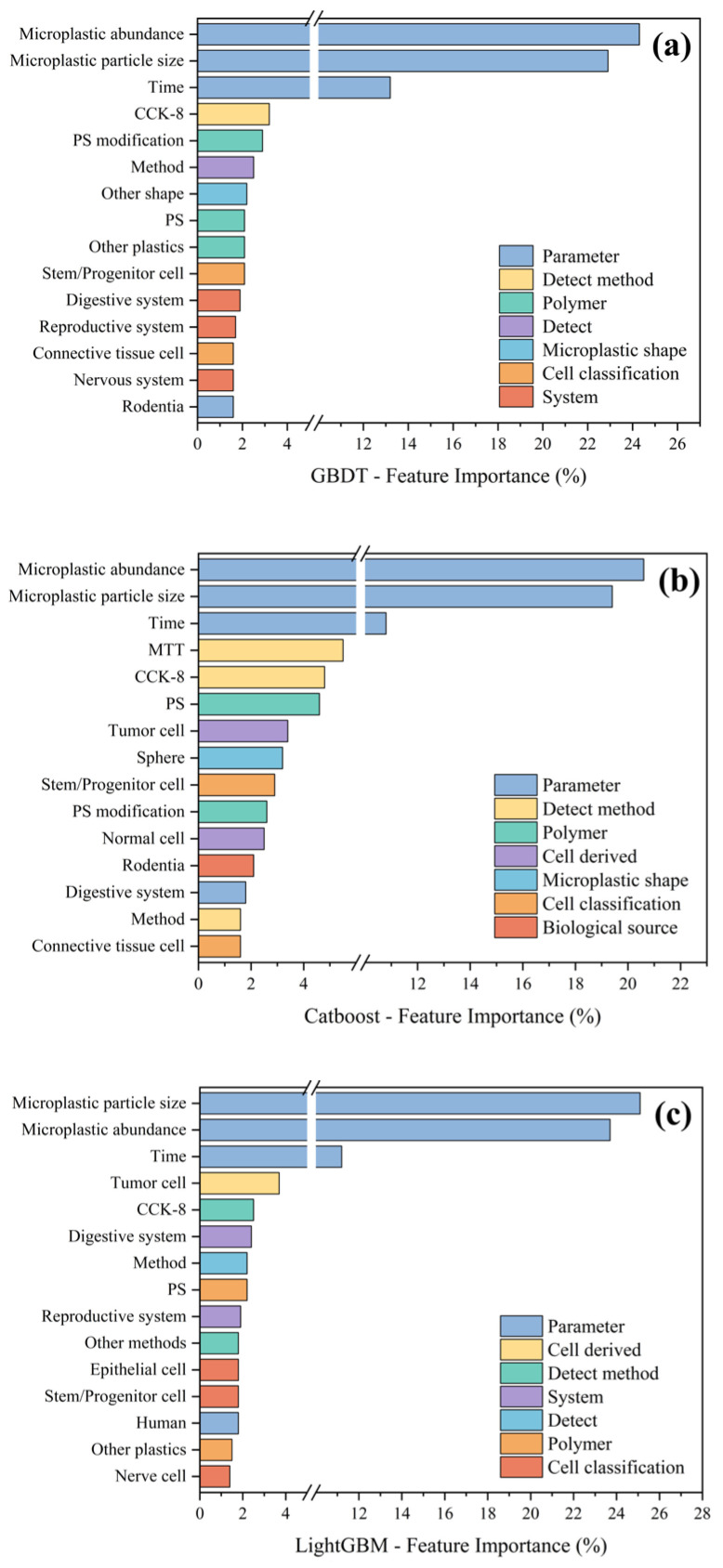
The distribution of feature importances for the three regression models: (**a**) GBDT, (**b**) CatBoost, and (**c**) LightGBM.

By integrating our model insights with the existing literature, we can further elucidate the mechanisms underlying microplastic-induced alterations in cell viability. First, particle size and dose emerge as critical determinants of cytotoxic potency [28], corroborating our earlier observations. Second, exposure duration also exerts a substantial effect. Among the three algorithms, categorical variables such as the assay method and polymer type rank highly in terms of feature importance, whereas cell-origin system and cell-type classification contribute minimally to predictive performance, as shown in Figure 12. Notably, microplastics of identical size and concentration produce divergent viability outcomes depending on surface chemistry, a mechanistic nuance captured by our feature-importance analyses. Thus, predictive models must incorporate multidimensional descriptors—size, concentration, shape, material composition, and surface properties—to accurately forecast cell viability. This requirement explains the superior performance of the GBDT model, whose capacity to model complex non-linear interactions among these factors enables a more faithful fit to the observed viability trends.

In summary, by applying machine learning models to cell viability data across diverse microplastic exposure conditions, this study demonstrates that GBDT, CatBoost, and LightGBM can all reliably predict microplastic-induced trends in cell viability, with GBDT performing marginally better. Variations in model performance can be ascribed to differences in each algorithm’s capacity to capture complex feature interactions and to mitigate overfitting. Crucially, the key determinants identified by our feature-importance analyses—namely the particle size, exposure dose, and surface characteristics of microplastics—are in accord with mechanistic insights reported in the toxicological literature. This concordance bolsters confidence in our predictive models, indicating that they not only deliver robust forecasting accuracy, but also, to a meaningful extent, embody the underlying biological processes.

#### 3.4.2. Machine Learning Classification Analysis of Cytotoxic Effects Under Microplastic Exposure Conditions

**Figure 13:**
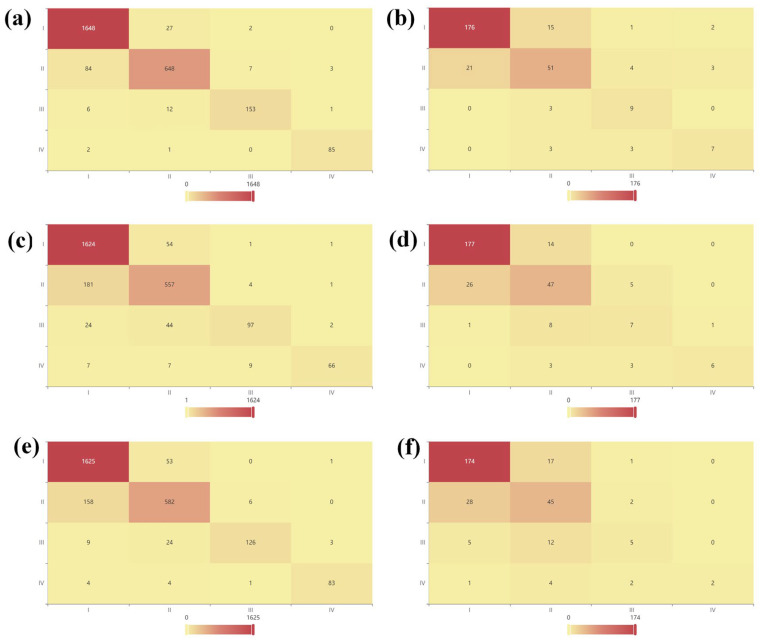
Confusion matrices for the three classification models: (**a**) GBDT on the training set, (**b**) GBDT on the test set, (**c**) CatBoost on the training set, (**d**) CatBoost on the test set, (**e**) LightGBM on the training set, and (**f**) LightGBM on the test set.

The performance metrics indicate that the GBDT model achieved a marginally higher accuracy, F_1_-score, and AUC than its counterparts, demonstrating a superior balance between positive and negative predictions, as well as superior overall discriminative capability. As shown by the confusion matrix in Figure 13, GBDT outperformed its counterparts on both the training and test sets: it attained an F_1_-score of 0.945 and an AUC of 0.998 on the training data, and values of 0.815 and 0.952, respectively, on the test data—clearly exceeding CatBoost (test F_1_ ≈ 0.789, AUC ≈ 0.945) and LightGBM (test F_1_ ≈ 0.743, AUC ≈ 0.933). These results underscore GBDT’s superior generalization performance for the cytotoxicity classification task.

**Figure 14:**
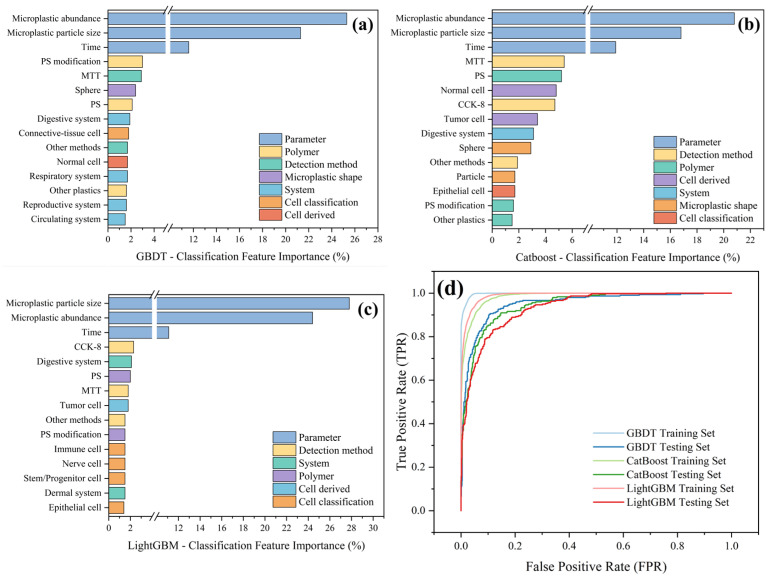
Feature-importance rankings and ROC–AUC curves for the three classification models: (**a**) GBDT, (**b**) CatBoost, and (**c**) LightGBM; (**d**) ROC–AUC curves.

In terms of model architecture, although CatBoost’s symmetric decision trees and ordered target encoding are reported to provide inherent balance, sensitivity to categorical variables, and resistance to overfitting [116], the GBDT model demonstrated superior performance in our study. Furthermore, feature-importance rankings in the classification setting closely paralleled those observed in the regression analysis. Notably, as shown in Figure 14d, GBDT achieved the largest area under the ROC curve on both the training and test sets, consistent with its overall predictive strength. In contrast, LightGBM, while attaining a higher AUC than CatBoost on the training set, underperformed compared to CatBoost on the test set, indicating overfitting and reduced generalization. CatBoost’s built-in overfitting detection and ordered boosting mechanisms conferred greater stability on unseen data. Future research should incorporate rigorous cross-validation and external validation cohorts to further evaluate model generalizability, and should leverage domain expertise in interpreting feature importances to uncover the fundamental drivers behind inter-model performance differences.

### 3.5. Technical Challenges and Future Directions

Prior to identification and quantification, microplastics must be efficiently separated from organic matter in human tissues. Unlike environmental matrices (e.g., water or soil), tissue samples demand reagents that combine high efficiency with analytical sensitivity. Geppner et al. demonstrated that common alkaline digestion agents (KOH/NaOH), pepsin, and pancreatin can effectively isolate 5 μm polystyrene microplastics from human blood [117]. Although spectroscopic and mass-spectrometric techniques (FTIR, Raman, Py-GC/MS) offer high sensitivity, matrix interference and variable recovery rates necessitate methodological standardization. Recent advances seek enhanced sensitivity and rapid throughput; for example, a strong correlation between the thermal diffusivity (D) of microplastic-spiked water samples and particle count (N) has enabled the development of a laser-assisted thermal-lens (TL) technique for precise microplastic quantification [118]. However, the complexity of real-world tissue matrices requires that analytical platforms demonstrate robust resistance to co-extracted interferents. These technological limitations substantially constrain accurate exposure and risk assessments: for instance, Py-GC/MS can report recovery rates ranging from 7% to 109% for blood microplastics, but non-specific pyrolysis by-products and co-eluting contaminants hinder reliable quantification of polyethylene and PVC abundances [119].

It should be noted that polystyrene (PS) is frequently used as a model compound in microplastic toxicity studies, and numerous investigations have confirmed the diverse biological toxicities of PS particles. However, in human tissues, PS is often not the predominant polymer detected; for example, polyethylene (PE) accounted for 53.6% of the microplastics found in actual thrombus samples [120]. This discrepancy likely reflects the limited ingestion of PS—since it is not commonly employed in food or beverage packaging—and underscores that microplastic properties such as density and surface chemistry influence both cellular interactions and transport dynamics. Therefore, researchers should consider selecting model microplastics that more closely mirror real-world exposures, rather than choosing polymers solely based on toxicity potency. In addition, systematic comparisons of toxicity across different polymer types, particle sizes, and surface modifications are needed, as well as investigations of chronic exposure effects at environmentally relevant concentrations. One often-overlooked issue is that laboratory procedures themselves can become sources of microplastic release—for instance, resin-embedded tissue specimens and samples prepared to study microplastic effects can shed particles during processing [121]. Thus, the development of techniques to reduce microplastic emissions from experimental resins is essential to minimize the environmental footprint of research activities.

Widespread detection of microplastics in human blood and their associations with diverse pathological processes underscore their importance as an emerging environmental risk factor. Systematic studies on topics ranging from molecular mechanisms to clinical phenotypes reveal that microplastics not only directly induce oxidative-stress-mediated damage and inflammation, but also amplify toxicity through organ crosstalk—via the gut–brain axis [85], the gut–liver axis [122], and the lung–brain axis [88]. Future research must integrate molecular toxicology, clinical medicine, and environmental engineering to construct predictive dose–response models and foster interdisciplinary collaboration for risk-based management strategies. Reducing dietary microplastic intake may be attainable by limiting consumption of high-accumulation foods (e.g., benthic organisms), but mitigating inhalation exposure is more complex. Although mask-wearing can block direct inhalation of airborne particles, the mass use of disposable masks during the COVID-19 pandemic inadvertently increased environmental microplastic loads due to improper disposal [123]. Furthermore, aged masks themselves release microplastics under sunlight exposure, posing inhalation risks during prolonged use [124]. Enhancing public awareness and the development of alternative materials are critical for reducing human microplastic exposure. Public health policies should prioritize minimizing detachable plastics in medical devices and food packaging, promote the application and validation of biodegradable polymers (e.g., PLA), and strengthen educational campaigns to curb everyday exposure. However, the ecological safety of biodegradable plastics must be comprehensively evaluated, since their degradation products can form microplastics with toxicity profiles comparable to those of conventional polymers. Moreover, health risk assessments of microplastics should evolve from single-polymer examinations to mixed-exposure scenarios, with attention directed to synergistic interactions involving per- and polyfluoroalkyl substances (PFOS, PFOA), endocrine-disrupting chemicals (EDCs), pharmaceuticals and personal care products (PPCPs), carcinogenic polycyclic aromatic hydrocarbons (PAHs), and brominated flame retardants. As globally mobile pollutants, microplastics demand coordinated international governance and policy implementation to effectively mitigate their human health impacts. However, we must remain aware that the interactions between microplastics and both biotic and abiotic environments are governed by multiple interrelated factors. Beyond those already studied, phenomena such as particle agglomeration and adsorption of biomolecules also warrant consideration in future investigations [125]. By leveraging established nanotoxicology evaluation systems alongside advanced instrumental analyses, the joint development of a comprehensive assessment framework may become a key research direction for the toxicity evaluation of human exposure to microplastics.

## 4. Conclusions

Since 2014, research on microplastics and human health has grown exponentially, expanding its focus from mere environmental pollution to human exposure and health outcomes. In this study, we combined meta-analysis with machine learning models to systematically assess the effects of microplastics on cell viability. Under the current study’s experimental conditions, cytotoxicity was predominantly limited to Grade I (62.8%) and Grade II (27.6%), indicating that most exposures induce only modest decreases in viability. However, when microplastic concentrations exceeded 40 μg/mL or particle diameters fell below 0.02 μm, cell viability declined significantly, reflecting an enhanced toxic response. Cells of reproductive and circulatory origin were the most sensitive, whereas connective-tissue cells exhibited the lowest survival rates, underscoring source-dependent sensitivity differences. We also identified an over-reliance on polystyrene (PS) and spherical particles in experimental systems, a bias that does not reflect the diverse polymer types and morphologies encountered in real-world exposures. By integrating multidimensional predictors—concentration, size, polymer chemistry, and exposure duration—our machine learning framework revealed quantitative relationships with cytotoxicity. Of the algorithms tested, the GBDT model performed best in both regression (test-set R^2^ = 0.737) and classification (accuracy = 81.5%), validating its ability to capture complex non-linear interactions and deliver robust predictions. Future work should expand the dataset to encompass more environmentally realistic parameters, such as mixed-polymer exposures, chronic low-dose regimens, and multicellular co-culture systems, and should further refine model architectures and feature-selection strategies to enhance predictive accuracy and generalizability. In addition, the toxic effects of microplastics go far beyond the scope of acute cytotoxicity. Long-term exposure can induce subchronic toxic effects such as chronic inflammatory response, persistent oxidative stress, DNA damage, and organelle dysfunction. Together, these mechanisms constitute potential cumulative health risks. Future research needs to draw on important experience in nanotoxicology: first, dosimetry should be strictly standardized and environmentally relevant concentrations used to avoid false-positive interference from high-dose exposure; second, attention should be paid to the modification of the surface properties of microplastics by biological matrices. Such interactions may affect their bioavailability and toxic potential.

## Figures and Tables

**Table 1:** Performance metrics of the machine learning regression models (the number of training-set data points was 2680, and the number of test-set data points was 298).

Model	Dataset	MSE	RMSE	MAE	MAPE	R^2^
GBDT	Training	68.607	8.283	5.649	7.539	0.87
	Test	134.118	11.581	7.6	10.026	0.737
CatBoost	Training	102.161	10.107	6.996	10.446	0.806
	Test	141.343	11.889	7.993	11.003	0.723
LightGBM	Training	122.372	11.062	7.478	10.103	0.759
	Test	203.459	14.264	9.459	13.918	0.702

**Table 2:** Performance metrics of the machine learning classification models (the number of training-set data points was 2680, and the number of test-set data points was 298).

Model	Dataset	Accuracy	Recall	Precision	F1	AUC
GBDT	Training	0.946	0.946	0.946	0.945	0.998
	Test	0.815	0.815	0.816	0.815	0.952
CatBoost	Training	0.875	0.875	0.874	0.87	0.984
	Test	0.795	0.795	0.788	0.789	0.945
LightGBM	Training	0.902	0.902	0.902	0.9	0.99
	Test	0.758	0.758	0.751	0.743	0.933

## Data Availability

Data will be made available on request.
